# Crystal structure of [3-(4,5-di­hydro-1,3-thia­zolin-2-yl-κ*N*)-1,3-thia­zolidine-2-thione-κ*S* ^2^](1,3-thia­zol­idine-2-thione-κ*S* ^2^)copper(I) nitrate

**DOI:** 10.1107/S160053681401544X

**Published:** 2014-07-19

**Authors:** Saowanit Saithong, Pirawan Klongkleaw, Chaveng Pakawatchai, Jedsada Mokakul

**Affiliations:** a Department of Chemistry and Center of Excellence for Innovation in Chemistry, Faculty of Science, Prince of Songkla University, Hat Yai, Songkhla 90112, Thailand

**Keywords:** copper(II) complex, 1,3-thia­zolidine-2-thione, 3-(2-thia­zolin-2-yl)thia­zolidine-2-thione, crystal structure

## Abstract

The mononuclear complex salt, [Cu(C_3_H_5_NS_2_)(C_6_H_8_N_2_S_3_)]NO_3_, contains a [C_9_H_13_CuN_3_S_5_]^+^ cation and an NO_3_^−^ anion. All of the non-H atoms of the cation lie on a mirror plane, as do the N and one O atom of the nitrate anion, such that the planes of the cation and anion are mutually orthogonal. The cationic complex adopts a slightly distorted trigonal–planar geometry about the Cu^I^ cation. In the crystal, layers parallel to (010) are generated by N—H⋯O hydrogen bonds, supported by short S⋯O [3.196 (4) and 3.038 (3) Å] and S⋯S contacts [3.4392 (13) Å]. Adjacent layers are linked by C—H⋯O hydrogen bonds and weak π–π stacking inter­actions [centroid–centroid distance = 4.0045 (10) Å] between the thia­zoline rings, forming a three-dimensional network. This stacking also imposes a close contact, of approximately 3.678 Å, between the Cu^I^ cations and the centroids of the six-membered chelate rings of mol­ecules in adjacent layers.

## Abstract 2

In the mononuclear complex title salt, all of the non-H atoms of the cation lie on a mirror plane, as do the N and one O atom of the nitrate anion, such that the planes of the cation and anion are mutually orthogonal. In the crystal, layers parallel to (010) are generated by N—H⋯O hydrogen bonds, supported by short S⋯O [3.196 (4) and 3.038 (3) Å] and S⋯S contacts [3.4392 (13) Å].

## Chemical context   

1,3-Thia­zolidine-2-thione (tzdSH: C_3_H_5_NS_2_), is a well known heterocyclic thione/thiol ligand. Crystallographic studies and investigations of its modes of coordination have been reported (Raper *et al.*, 1998 ▶; Ainscough *et al.*, 1985 ▶; Kubiak & Głowiak, 1987 ▶; Cowie & Sielisch, 1988 ▶; Ballester *et al.*, 1992 ▶; Fackler *et al.*, 1992 ▶; Saithong *et al.*, 2007 ▶). We are inter­ested in the coordination behaviour and structure of tzdSH complexes with Cu^II^ cations. We have normally used Cu(NO_3_)_2_·3H_2_O as the starting material with the possibility that the NO_3_^−^ anions could function as simple counter-ions to balance the charge on the metal or alternatively act as a ligand to the metal ion (Ferrer *et al.*, 2000 ▶; Pal *et al.*, 2005 ▶; Khavasi *et al.*, 2011 ▶). However, the tzdSH ligand could also act as a reducing agent during the reaction, converting Cu^II^ to Cu^I^ and forming 3-(2-thia­zolin-2-yl)thia­zolidine-2-thione [tztzdt or C_6_H_8_N_2_S_3_] in the process. A similar reduction reaction was described previously by Ainscough *et al.* (1985 ▶). Complexation of the resulting Cu^I^ cation to both the tztzdt ligand thus formed, and to tzdSH generates the title compound.
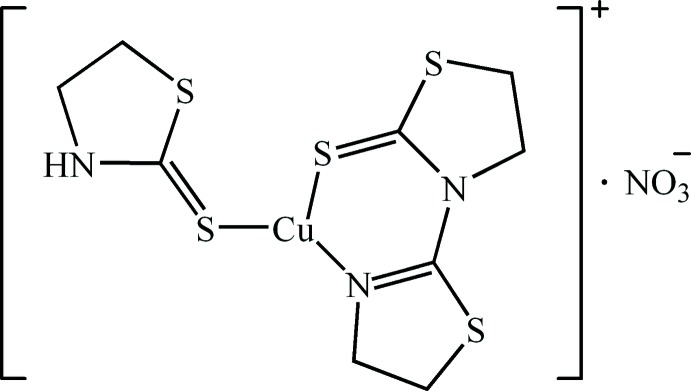


## Structural commentary   

**Figure 1:**
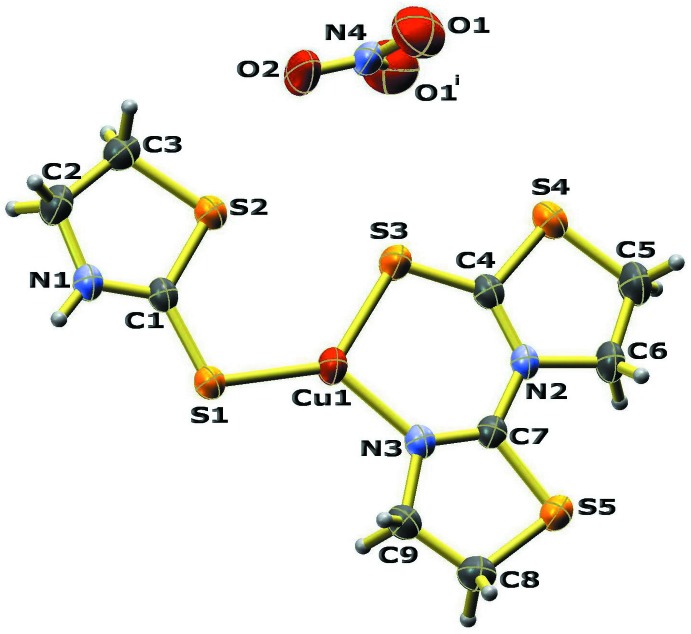
Mol­ecular structure of the title compound, with displacement ellipsoids drawn at the 30% probability level. [Symmetry code: (i) *x*, −*y* + ½, *z*.]

The title compound is a mononuclear Cu^I^ complex and its structure is shown in Fig. 1 ▶. The Cu^I^ atom has a distorted trigonal–planar coordination geometry and is chelated by the exocyclic S3 atom and the N3 atom of the thia­zolidine ring of the tztzdt ligand, forming a six-membered chelate ring. The trigonal coordination sphere is completed by the exocyclic S1 atom of the tzdSH ligand. The NO_3_^−^ acts solely as counter-ion. The complex mol­ecule is strictly planar as all non-hydrogen atoms of the complex lie on a mirror plane. Atoms N4 and O1 of the nitrate counter-ion also lie on a mirror plane, such that the mirror plane of the cationic complex is perpendicular to that of the NO_3_^−^ anion. The Cu1—S1 [2.1774 (9) Å], and Cu1—N3 [1.956 (3) Å] bond lengths are not unusual in comparison with the mean values [Cu—S = 2.21 (3) and Cu—N = 1.99 (3) Å] found in the Cambridge Structural Database. In contrast, the Cu1—S1 distance of 2.1774 (9) Å is somewhat shorter than those previously reported for other Cu^I^ complexes of tzdSH [mean Cu—S = 2.33 (1) Å].

## Supra­molecular features   

**Figure 2:**
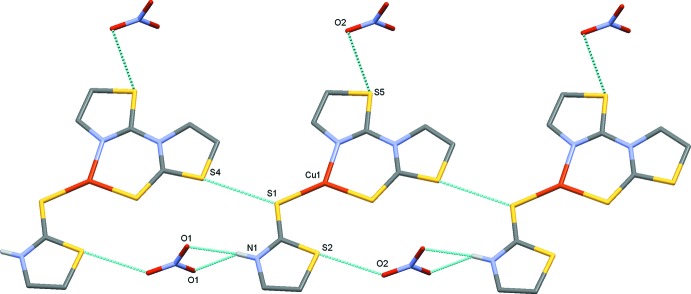
Two-dimensional sheets of mol­ecules parallel to (010). Hydrogen bonds are drawn as dashed lines and H atoms not involved in hydrogen bonding have been omitted for clarity.

**Figure 3:**
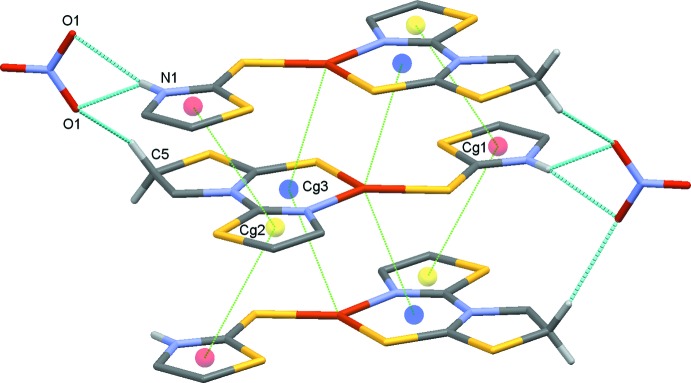
π–π stacking inter­actions between mol­ecules. Centroid–centroid and unusual Cu1–centroid contacts are drawn as dotted lines with the centroids shown as coloured spheres. Hydrogen bonds are drawn as dashed lines and H atoms not involved in hydrogen bonding have been omitted for clarity.

**Figure 4:**
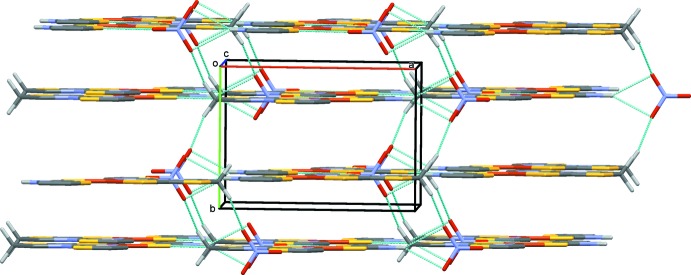
The overall packing of the title compound. Hydrogen bonds are drawn as dashed lines and H atoms not involved in hydrogen bonding have been omitted for clarity.

In the crystal, layers are generated parallel to (010) by classical N1—H1⋯O1 hydrogen bonds (Table 1 ▶) supported by short S2⋯O2 [3.038 (3) Å], S5⋯O2 [3.196 (4) Å] and S1⋯S4 [3.4392 (13) Å] contacts (Fig. 2 ▶). Adjacent layers are linked by C5—H5*A*⋯O1 hydrogen bonds and weak π–π stacking inter­actions [centroid–centroid distance 4.0045 (10) Å] between the thia­zoline rings of a tzdSH ligand with those of tztzdt ligands in adjacent layers, forming a three-dimensional network. This stacking also imposes a close contact, of approximately 3.678 Å, between the copper cations and the centroids of the six-membered Cu1, S3, C4, N2, C7, N3 chelate rings of the mol­ecules in the adjacent layers (Fig. 3 ▶). The three-dimensional network of stacked layers is shown in Fig. 4 ▶.

## Database survey   

Only four discrete reports are given of transition-metal complexes with the metal atom chelated by the tztzdt ligand. All are copper complexes, three of Cu^I^ (Lobana *et al.*, 2013 ▶) and the fourth a Cu^II^ coordination polymer (Ainscough *et al.*, 1985 ▶). Complexes of tzdSH are more plentiful with 29 unique entries, ten of which involve Cu^I^ cations (see, for example: Lobana *et al.*, 2013 ▶; Raper *et al.*, 1998 ▶).

## Synthesis and crystallization   

1,3-Thia­zolidine-2-thione (0.1 g, 0.084 mmol) was added to a solution of Cu(NO_3_)_2_·3H_2_O (0.07 g, 0.039 mmol) in an MeOH: EtOH solvent mixture (1/1 *v*/*v*) at 340 K. The mixture was refluxed for 5 h. Rod-like yellow crystals appeared after the light-brown filtrate had been kept at room temperature for a day (yield 10%). The crystals melt and decompose at 434–436 K.

## Refinement   

The N1—H1 hydrogen atom was located in a difference map and its coordinates were refined with *U*_iso_(H) = 1.2*U*_eq_(N). The hydrogen atoms of the methyl­ene groups were positioned geometrically and allowed to ride on their parent atoms, with *d*(C—H) = 0.97 Å and *U*_iso_ = 1.2*U*_eq_(C). Experimental details are given in Table 2 ▶.

## Supplementary Material

Crystal structure: contains datablock(s) I, pk030-p21-m. DOI: 10.1107/S160053681401544X/sj5420sup1.cif

Structure factors: contains datablock(s) I. DOI: 10.1107/S160053681401544X/sj5420Isup2.hkl

CCDC reference: 1011568

Additional supporting information:  crystallographic information; 3D view; checkCIF report

## Figures and Tables

**Table 1:** Hydrogen-bond geometry (Å, °)

*D*—H⋯*A*	*D*—H	H⋯*A*	*D*⋯*A*	*D*—H⋯*A*
N1—H1⋯O1^i^	0.84 (2)	2.25 (2)	2.981 (4)	146 (2)
N1—H1⋯O1^ii^	0.84 (2)	2.25 (2)	2.981 (4)	146 (2)
C5—H5*A*⋯O1^iii^	0.97	2.62	3.388 (4)	136

Symmetry codes: (i) 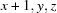; (ii) 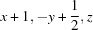; (iii) 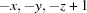.

**Table 2:** Experimental details

Crystal data	
Chemical formula	[Cu(C_3_H_5_NS_2_)(C_6_H_8_N_2_S_3_)]NO_3_
*M*_r_	449.13
Crystal system, space group	Monoclinic, *P*2_1_/*m*
Temperature (K)	293
*a*, *b*, *c* (Å)	9.8937 (6), 6.9932 (5), 11.8054 (7)
β (°)	102.078 (1)
*V* (Å^3^)	798.72 (9)
*Z*	2
Radiation type	Mo *K*α
μ (mm^−1^)	2.04
Crystal size (mm)	0.35 × 0.12 × 0.06

Data collection	
Diffractometer	Bruker *APEX* CCD area detector
Absorption correction	Multi-scan (*SADABS*; Bruker, 2003 ▶)
*T*_min_, *T*_max_	0.814, 1.000
No. of measured, independent and observed [*I* > 2s(*I*)] reflections	8700, 1534, 1402
*R*_int_	0.026
(sin θ/λ)_max_ (Å^−1^)	0.595

Refinement	
*R*[*F* ^2^ > 2σ(*F* ^2^)], *wR*(*F* ^2^), *S*	0.029, 0.080, 1.07
No. of reflections	1534
No. of parameters	133
No. of restraints	1
H-atom treatment	H atoms treated by a mixture of independent and constrained refinement
Δρ_max_, Δρ_min_ (e Å^−3^)	0.37, −0.27

Computer programs: *SMART* (Bruker, 1998 ▶), *SAINT* (Bruker, 2003 ▶), *ShelXle* (Hübschle *et al.*, 2011 ▶), *SHELXTL* (Sheldrick, 2008 ▶), *Mercury* (Macrae *et al.*, 2008 ▶), *PLATON* (Spek, 2009 ▶) and *publCIF* (Westrip, 2010 ▶).

## supplementary crystallographic information

### Crystal data

**Table:**

[Cu(C_3_H_5_NS_2_)(C_6_H_8_N_2_S_3_)]NO_3_	*F*(000) = 456
*M**_r_* = 449.13	*D*_x_ = 1.867 Mg m^−^^3^
Monoclinic, *P*2_1_/*m*	Mo *K*α radiation, λ = 0.71073 Å
Hall symbol: -P 2yb	Cell parameters from 3943 reflections
*a* = 9.8937 (6) Å	θ = 2.5–28.0°
*b* = 6.9932 (5) Å	µ = 2.04 mm^−^^1^
*c* = 11.8054 (7) Å	*T* = 293 K
β = 102.078 (1)°	Block, yellow
*V* = 798.72 (9) Å^3^	0.35 × 0.12 × 0.06 mm
*Z* = 2	

### Data collection

**Table:**

Bruker APEX CCD area-detector diffractometer	1534 independent reflections
Radiation source: fine-focus sealed tube	1402 reflections with *I* > 2s(*I*)
Graphite monochromator	*R*_int_ = 0.026
Frames, each covering 0.3 ° in ω scans	θ_max_ = 25.0°, θ_min_ = 1.8°
Absorption correction: multi-scan (*SADABS*; Bruker, 2003)	*h* = −11→11
*T*_min_ = 0.814, *T*_max_ = 1.000	*k* = −8→8
8700 measured reflections	*l* = −14→14

### Refinement

**Table:**

Refinement on *F*^2^	Secondary atom site location: difference Fourier map
Least-squares matrix: full	Hydrogen site location: inferred from neighbouring sites
*R*[*F*^2^ > 2σ(*F*^2^)] = 0.029	H atoms treated by a mixture of independent and constrained refinement
*wR*(*F*^2^) = 0.080	*w* = 1/[σ^2^(*F*_o_^2^) + (0.0471*P*)^2^ + 0.3435*P*] where *P* = (*F*_o_^2^ + 2*F*_c_^2^)/3
*S* = 1.07	(Δ/σ)_max_ = 0.001
1534 reflections	Δρ_max_ = 0.37 e Å^−^^3^
133 parameters	Δρ_min_ = −0.27 e Å^−^^3^
1 restraint	Extinction correction: *SHELXTL* (Sheldrick, 2008), Fc^*^=kFc[1+0.001xFc^2^λ^3^/sin(2θ)]^-1/4^
Primary atom site location: structure-invariant direct methods	Extinction coefficient: 0.0074 (13)

### Special details

**Table:**

Geometry. All e.s.d.'s (except the e.s.d. in the dihedral angle between two l.s. planes) are estimated using the full covariance matrix. The cell e.s.d.'s are taken into account individually in the estimation of e.s.d.'s in distances, angles and torsion angles; correlations between e.s.d.'s in cell parameters are only used when they are defined by crystal symmetry. An approximate (isotropic) treatment of cell e.s.d.'s is used for estimating e.s.d.'s involving l.s. planes.
Refinement. Refinement of *F*^2^ against all reflections. The weighted *R*-factor *wR* and goodness of fit *S* are based on *F*^2^, conventional *R*-factors *R* are based on *F*, with *F* set to zero for negative *F*^2^. The threshold expression of *F*^2^ > σ(*F*^2^) is used only for calculating *R*-factors(gt) *etc*. and is not relevant to the choice of reflections for refinement. *R*-factors based on *F*^2^ are statistically about twice as large as those based on *F*, and *R*-factors based on al data will be even larger.

### Fractional atomic coordinates and isotropic or equivalent isotropic displacement parameters (Å^2^)

**Table:**

	*x*	*y*	*z*	*U*_iso_*/*U*_eq_	Occ. (<1)
Cu1	0.53707 (4)	0.2500	0.52014 (4)	0.05377 (19)	
S1	0.75417 (8)	0.2500	0.60684 (7)	0.0471 (2)	
S2	0.61224 (9)	0.2500	0.80799 (8)	0.0494 (2)	
S3	0.34723 (10)	0.2500	0.58878 (8)	0.0641 (3)	
S4	0.05567 (9)	0.2500	0.50537 (9)	0.0571 (3)	
S5	0.28736 (10)	0.2500	0.15159 (7)	0.0527 (3)	
N1	0.8731 (3)	0.2500	0.8311 (3)	0.0509 (7)	
H1	0.951 (3)	0.2500	0.813 (3)	0.061*	
N2	0.2147 (3)	0.2500	0.3586 (2)	0.0404 (6)	
N3	0.4543 (3)	0.2500	0.3544 (2)	0.0446 (6)	
C1	0.7591 (3)	0.2500	0.7514 (3)	0.0408 (7)	
C2	0.7099 (4)	0.2500	0.9567 (3)	0.0565 (9)	
H2A	0.6879	0.3626	0.9973	0.068*	0.50
H2B	0.6879	0.1374	0.9973	0.068*	0.50
C3	0.8615 (4)	0.2500	0.9509 (3)	0.0654 (11)	
H3A	0.9066	0.3624	0.9897	0.079*	0.50
H3B	0.9066	0.1376	0.9897	0.079*	0.50
C4	0.2171 (3)	0.2500	0.4737 (3)	0.0434 (7)	
C5	−0.0321 (4)	0.2500	0.3563 (4)	0.0737 (12)	
H5A	−0.0901	0.1375	0.3396	0.088*	0.50
H5B	−0.0901	0.3625	0.3396	0.088*	0.50
C6	0.0736 (3)	0.2500	0.2847 (3)	0.0592 (10)	
H6A	0.0618	0.3623	0.2354	0.071*	0.50
H6B	0.0618	0.1377	0.2354	0.071*	0.50
C7	0.3273 (3)	0.2500	0.3040 (3)	0.0392 (7)	
C8	0.4694 (4)	0.2500	0.1485 (3)	0.0577 (9)	
H8A	0.4926	0.1374	0.1085	0.069*	0.50
H8B	0.4926	0.3626	0.1085	0.069*	0.50
C9	0.5473 (4)	0.2500	0.2725 (3)	0.0542 (9)	
H9A	0.6062	0.1379	0.2861	0.065*	0.50
H9B	0.6062	0.3621	0.2861	0.065*	0.50
N4	0.2237 (3)	0.2500	0.8549 (2)	0.0596 (9)	
O1	0.1564 (3)	0.1019 (4)	0.8343 (2)	0.1071 (8)	
O2	0.3440 (3)	0.2500	0.8948 (3)	0.1254 (18)	

### Atomic displacement parameters (Å^2^)

**Table:**

	*U*^11^	*U*^22^	*U*^33^	*U*^12^	*U*^13^	*U*^23^
Cu1	0.0365 (3)	0.0762 (4)	0.0445 (3)	0.000	−0.00081 (18)	0.000
S1	0.0334 (4)	0.0643 (5)	0.0435 (5)	0.000	0.0076 (3)	0.000
S2	0.0366 (4)	0.0644 (5)	0.0474 (5)	0.000	0.0093 (4)	0.000
S3	0.0403 (5)	0.1106 (8)	0.0396 (5)	0.000	0.0045 (4)	0.000
S4	0.0369 (5)	0.0729 (6)	0.0632 (6)	0.000	0.0145 (4)	0.000
S5	0.0501 (5)	0.0668 (6)	0.0383 (4)	0.000	0.0025 (4)	0.000
N1	0.0345 (15)	0.0683 (19)	0.0471 (16)	0.000	0.0020 (12)	0.000
N2	0.0313 (13)	0.0443 (14)	0.0427 (15)	0.000	0.0010 (11)	0.000
N3	0.0350 (14)	0.0541 (16)	0.0438 (15)	0.000	0.0059 (11)	0.000
C1	0.0339 (16)	0.0407 (16)	0.0461 (18)	0.000	0.0046 (13)	0.000
C2	0.060 (2)	0.066 (2)	0.0425 (18)	0.000	0.0085 (16)	0.000
C3	0.053 (2)	0.091 (3)	0.047 (2)	0.000	−0.0020 (17)	0.000
C4	0.0358 (16)	0.0442 (17)	0.0495 (18)	0.000	0.0076 (14)	0.000
C5	0.039 (2)	0.110 (4)	0.068 (3)	0.000	0.0015 (18)	0.000
C6	0.0361 (18)	0.080 (3)	0.055 (2)	0.000	−0.0037 (16)	0.000
C7	0.0390 (17)	0.0391 (16)	0.0380 (16)	0.000	0.0044 (13)	0.000
C8	0.055 (2)	0.069 (2)	0.052 (2)	0.000	0.0168 (17)	0.000
C9	0.0401 (18)	0.072 (2)	0.052 (2)	0.000	0.0131 (15)	0.000
N4	0.0409 (17)	0.101 (3)	0.0340 (15)	0.000	0.0022 (12)	0.000
O1	0.110 (2)	0.0932 (18)	0.122 (2)	−0.0237 (16)	0.0328 (17)	−0.0061 (17)
O2	0.0409 (16)	0.273 (6)	0.0588 (18)	0.000	0.0021 (14)	0.000

### Geometric parameters (Å, º)

**Table:**

Cu1—N3	1.956 (3)	C2—C3	1.516 (5)
Cu1—S1	2.1774 (9)	C2—H2A	0.9700
Cu1—S3	2.1957 (10)	C2—H2B	0.9700
S1—C1	1.698 (3)	C3—H3A	0.9700
S2—C1	1.721 (3)	C3—H3B	0.9700
S2—C2	1.818 (4)	C5—C6	1.475 (6)
S3—C4	1.665 (3)	C5—H5A	0.9700
S4—C4	1.715 (3)	C5—H5B	0.9700
S4—C5	1.792 (4)	C6—H6A	0.9700
S5—C7	1.760 (3)	C6—H6B	0.9700
S5—C8	1.810 (4)	C8—C9	1.505 (5)
N1—C1	1.308 (4)	C8—H8A	0.9700
N1—C3	1.443 (5)	C8—H8B	0.9700
N1—H1	0.841 (19)	C9—H9A	0.9700
N2—C4	1.354 (4)	C9—H9B	0.9700
N2—C7	1.398 (4)	N4—O2	1.185 (4)
N2—C6	1.483 (4)	N4—O1	1.228 (3)
N3—C7	1.273 (4)	N4—O1^i^	1.228 (3)
N3—C9	1.468 (4)		
			
N3—Cu1—S1	129.45 (8)	N2—C4—S4	113.4 (2)
N3—Cu1—S3	99.07 (8)	S3—C4—S4	114.7 (2)
S1—Cu1—S3	131.48 (4)	C6—C5—S4	107.9 (3)
C1—S1—Cu1	106.90 (11)	C6—C5—H5A	110.1
C1—S2—C2	93.06 (16)	S4—C5—H5A	110.1
C4—S3—Cu1	105.89 (12)	C6—C5—H5B	110.1
C4—S4—C5	93.88 (18)	S4—C5—H5B	110.1
C7—S5—C8	90.55 (16)	H5A—C5—H5B	108.4
C1—N1—C3	118.1 (3)	C5—C6—N2	110.9 (3)
C1—N1—H1	121 (3)	C5—C6—H6A	109.5
C3—N1—H1	121 (3)	N2—C6—H6A	109.5
C4—N2—C7	127.9 (3)	C5—C6—H6B	109.5
C4—N2—C6	114.0 (3)	N2—C6—H6B	109.5
C7—N2—C6	118.1 (3)	H6A—C6—H6B	108.1
C7—N3—C9	112.7 (3)	N3—C7—N2	126.0 (3)
C7—N3—Cu1	129.3 (2)	N3—C7—S5	117.8 (2)
C9—N3—Cu1	118.0 (2)	N2—C7—S5	116.2 (2)
N1—C1—S1	124.2 (3)	C9—C8—S5	106.8 (2)
N1—C1—S2	113.1 (2)	C9—C8—H8A	110.4
S1—C1—S2	122.75 (18)	S5—C8—H8A	110.4
C3—C2—S2	106.7 (3)	C9—C8—H8B	110.4
C3—C2—H2A	110.4	S5—C8—H8B	110.4
S2—C2—H2A	110.4	H8A—C8—H8B	108.6
C3—C2—H2B	110.4	N3—C9—C8	112.1 (3)
S2—C2—H2B	110.4	N3—C9—H9A	109.2
H2A—C2—H2B	108.6	C8—C9—H9A	109.2
N1—C3—C2	109.0 (3)	N3—C9—H9B	109.2
N1—C3—H3A	109.9	C8—C9—H9B	109.2
C2—C3—H3A	109.9	H9A—C9—H9B	107.9
N1—C3—H3B	109.9	O2—N4—O1	122.47 (18)
C2—C3—H3B	109.9	O2—N4—O1^i^	122.47 (18)
H3A—C3—H3B	108.3	O1—N4—O1^i^	115.0 (4)
N2—C4—S3	131.9 (3)		
			
N3—Cu1—S1—C1	180.0	Cu1—S3—C4—S4	180.0
S3—Cu1—S1—C1	0.0	C5—S4—C4—N2	0.0
N3—Cu1—S3—C4	0.0	C5—S4—C4—S3	180.0
S1—Cu1—S3—C4	180.0	C4—S4—C5—C6	0.0
S1—Cu1—N3—C7	180.0	S4—C5—C6—N2	0.0
S3—Cu1—N3—C7	0.0	C4—N2—C6—C5	0.0
S1—Cu1—N3—C9	0.0	C7—N2—C6—C5	180.0
S3—Cu1—N3—C9	180.0	C9—N3—C7—N2	180.0
C3—N1—C1—S1	180.0	Cu1—N3—C7—N2	0.0
C3—N1—C1—S2	0.0	C9—N3—C7—S5	0.0
Cu1—S1—C1—N1	180.0	Cu1—N3—C7—S5	180.0
Cu1—S1—C1—S2	0.0	C4—N2—C7—N3	0.0
C2—S2—C1—N1	0.0	C6—N2—C7—N3	180.0
C2—S2—C1—S1	180.0	C4—N2—C7—S5	180.0
C1—S2—C2—C3	0.0	C6—N2—C7—S5	0.0
C1—N1—C3—C2	0.000 (1)	C8—S5—C7—N3	0.0
S2—C2—C3—N1	0.000 (1)	C8—S5—C7—N2	180.0
C7—N2—C4—S3	0.0	C7—S5—C8—C9	0.0
C6—N2—C4—S3	180.0	C7—N3—C9—C8	0.0
C7—N2—C4—S4	180.0	Cu1—N3—C9—C8	180.0
C6—N2—C4—S4	0.0	S5—C8—C9—N3	0.0
Cu1—S3—C4—N2	0.0		

Symmetry code: (i) *x*, −*y*+1/2, *z*.

### Hydrogen-bond geometry (Å, º)

**Table:**

*D*—H···*A*	*D*—H	H···*A*	*D*···*A*	*D*—H···*A*
N1—H1···O1^ii^	0.84 (2)	2.25 (2)	2.981 (4)	146 (2)
N1—H1···O1^iii^	0.84 (2)	2.25 (2)	2.981 (4)	146 (2)
C5—H5*A*···O1^iv^	0.97	2.62	3.388 (4)	136

Symmetry codes: (ii) *x*+1, *y*, *z*; (iii) *x*+1, −*y*+1/2, *z*; (iv) −*x*, −*y*, −*z*+1.
